# Monitoring of Interfractional Proton Range Verification and Dosimetric Impact Based on Daily CBCT for Pediatric Patients with Pelvic Tumors

**DOI:** 10.3390/cancers15174200

**Published:** 2023-08-22

**Authors:** Ozgur Ates, Jinsoo Uh, Fakhriddin Pirlepesov, Chia-Ho Hua, Thomas E. Merchant, Matthew J. Krasin

**Affiliations:** St. Jude Children’s Research Hospital, Memphis, TN 38105, USA; jinsoo.uh@stjude.org (J.U.); fakhriddin.pirlepesov@stjude.org (F.P.); chia-ho.hua@stjude.org (C.-H.H.); thomas.merchant@stjude.org (T.E.M.); matthew.krasin@stjude.org (M.J.K.)

**Keywords:** proton range uncertainty, water equivalent path length, pediatric pelvic tumors, interfractional range verification, synthetic CT

## Abstract

**Simple Summary:**

The research highlights the application of synthetic CT images, which are created by deforming planning CT scans to match daily CBCT anatomy in interfractional proton therapy for pediatric patients with pelvic tumors. The objective of the study is to identify changes in the proton path length and determine the impact of anatomical changes on the treatment plan’s quality. The findings reveal that the water equivalent path length method can effectively estimate proton range deviations on synthetic-CT images. The daily synthetic CT images can also be utilized as a surrogate to calculate dose and predict dosimetric changes in the plan of the day. This approach eliminates the need for frequent rescanning, thereby making the adaptive therapy process more streamlined and less burdensome for young patients. The results have the potential to improve the precision of proton therapy, hence paving the way for more effective treatments.

**Abstract:**

(1) Background: Synthetic CT images of the pelvis were generated from daily CBCT images to monitor changes in water equivalent path length (WEPL) and determine the dosimetric impact of anatomy changes along the proton beam’s path; (2) Methods: Ten pediatric patients with pelvic tumors treated using proton therapy with daily CBCT were included. The original planning CT was deformed to the same-day CBCT to generate synthetic CT images for WEPL comparison and dosimetric evaluation; (3) Results: WEPL changes of 20 proton fields at the distal edge of the CTV ranged from 0.1 to 12 mm with a median of 2.5 mm, and 75th percentile of 5.1 mm for (the original CT—rescanned CT) and ranged from 0.3 to 10.1 mm with a median of 2.45 mm and 75th percentile of 4.8 mm for (the original CT—synthetic CT). The dosimetric impact was due to proton range pullback or overshoot, which led to reduced coverage in CTV Dmin averaging 12.1% and 11.3% in the rescanned and synthetic CT verification plans, respectively; (4) Conclusions: The study demonstrated that synthetic CT generated by deforming the original planning CT to daily CBCT can be used to quantify proton range changes and predict adverse dosimetric scenarios without the need for excessive rescanned CT scans during large interfractional variations in adaptive proton therapy of pediatric pelvic tumors.

## 1. Introduction

Intensity-modulated proton therapy (IMPT) is currently the most advanced proton delivery technique to create a highly conformal dose distribution to tumors while sparing adjacent normal tissues; however, interfractional anatomical variations along the proton beam path can adversely impact dosimetry leading to an insufficient dose to the tumor and an unplanned dose to normal tissue [1].

To address this challenge, water equivalent path length (WEPL) analysis has been developed to accurately determine the path length of the proton beam through different tissues. WEPL is defined as the distance a proton would travel through water to reach a given point in a tissue. By using WEPL analysis, the energy of the proton beam can be adjusted to ensure that the tumor receives the appropriate dose of radiation while minimizing damage to surrounding healthy tissues [2]. Several techniques are used to measure WEPL, including range measurements and diode detectors [3]. Monte Carlo simulations are also used to model the behavior of protons as they interact with different tissues [4].

Image-Guided Radiation Therapy (IGRT) has emerged as a cornerstone in modern radiation oncology, enabling clinicians to adapt treatment plans to the dynamic anatomical changes that patients undergo during the course of radiotherapy [5]. However, the dependence on daily imaging for precise targeting introduces challenges in terms of both resource utilization and patient comfort. One innovative solution to address these challenges is the creation of synthetic CT images from daily IGRT images [6]. Synthetic CT is a computational technique that leverages deformable image registration methods to generate high-fidelity CT-like images from different imaging modalities, such as CBCT images. These synthetic CT images offer a representation of the patient’s anatomy at each treatment session without the need for repeated CT scans, minimizing patient exposure to ionizing radiation, and reducing the burden on imaging resources.

Furthermore, while IGRT has been used as a standard practice to address interfractional variations by repositioning the patient based on CBCT images acquired immediately before radiation therapy (RT) delivery [7], it is important to note that IGRT repositioning techniques do not adequately address interfractional uncertainties such as body circumference changes, relative geometric changes between targets and organs at risk (OAR), and anatomical changes (e.g., deformations) throughout the course of proton therapy, if left uncorrected, may reduce the probability of tumor control [8].

In pediatric patients changes in superficial or deep soft tissues may occur due to weight gain/loss, systemic steroids administration, or IV hydration for chemotherapy resulting in a significant impact on plan quality during radiation therapy [9]. WEPL analysis could be an ideal and practical verification method for the changes of weight gain or loss, owing to homogenous expansion or contraction of the external body contour. By allowing for early detection and correction of changes in body contour and tissue distribution, WEPL analysis can contribute to improved treatment efficacy and patient outcomes.

In this study, we employed the WEPL method as a surrogate measure for proton range verification and examined the impact on the plan quality of pediatric pelvic treatments. To date, no studies have been published assessing interfractional WEPL in pediatric patients receiving pencil beam scanning proton therapy to pelvic disease sites.

## 2. Materials and Methods

In the methods section, the design of WEPL calculation, synthetic CT generation and the phantom validation is explained in Section 2.1. Patient selection and image data are described in Section 2.2. Treatment planning aspects are outlined in Section 2.3.

### 2.1. WEPL Calculation, Synthetic-CT Generation and Phantom Validation

The WEPL was calculated up to the distal surface of clinical target volume (CTV) via the linear integration of relative stopping proton stopping power (RPSP) using a stoichiometric calibration curve [10]. The RPSP values were linearly integrated per voxel along with the beam’s eye view (BEV) direction from patient’s external body surface to the distal surface of CTV [11,12]. The generated 2D map of distal surface of CTV was called WEPL map.

**Figure 1:**
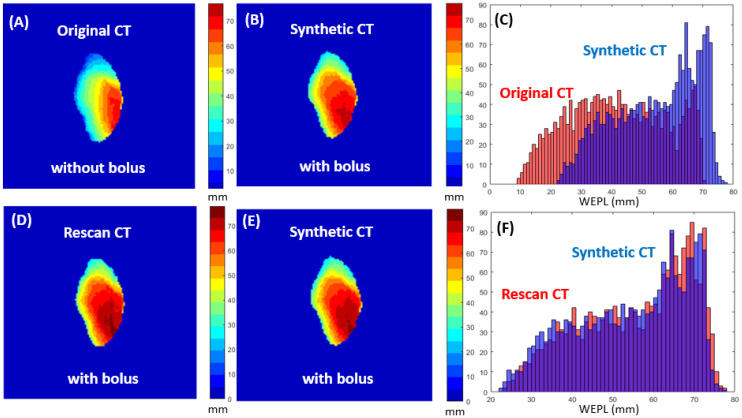
2D distal projection of CTV was calculated on original CT without bolus (**A**), synthetic CT with bolus based on CBCT anatomy (**B**), and the difference was found as 10.1 ± 1.1 mm (mean ± SD) between the two in terms of WEPL (**C**). 2D distal projection of the same CTV was calculated on rescanned CT with bolus (**D**), synthetic CT with bolus based on CBCT anatomy (**E**), and the difference was found as 0.1 ± 0.2 mm (mean ± SD) between the two in terms of WEPL (**F**).

Phantom validation was conducted to verify the accuracy of WEPL calculation. An anthropomorphic end-to-end verification ‘STEEV’ phantom (CIRS Inc., Norfolk, VA, USA) was used to simulate a scenario of 1 cm of weight gain by applying a bolus of 1 cm thickness to the surface of the phantom’s external body. STEEV phantom was first imaged with diagnostic CT using Philips IQon Spectral CT (Philips Healthcare, Cleveland, OH, USA) with and without bolus. Secondly, the STEEV phantom was scanned with bolus using an in-room robotic CBCT device (Hitachi, Ltd., Tokyo, Japan) for image-guided radiation therapy purposes. A commercial image manipulation ‘MIM’ software version 7.3.2 (MIM Software Inc., Cleveland, OH, USA) was used to deform CT images without bolus by generating synthetic-CT images based on CBCT anatomy with bolus. WEPL maps were generated to compare any range differences between the original CT without bolus and synthetic-CT with bolus and also the rescanned CT with bolus and synthetic CT with bolus as shown in Figure 1.

### 2.2. The Patient Selection and Image Data

Ten pelvis patients who had previously undergone a course of pediatric proton therapy and CT-simulated more than once due to external tissue discrepancies were selected in this retrospective study. Institutional Review Board (IRB) approval was obtained prior to analysis. The imaging data of the original and rescanned CT were acquired on 10 patients using a Philips IQon spectral CT with the clinical helical scan protocol of pelvis (120 kVp, auto collimation, 500 mm field of view, 512 × 512 matrix size, and Dose Right Index of 20). Synthetic CT images were created on the same day as the rescanned CT images to keep the anatomy similar during deformable image registration of daily CBCT images using a normalized intensity-based algorithm. Table 1 outlines the patients with diagnoses, age, sex, treatment site, fractionation, beam orientation, and the number of CT rescans that the patients were simulated during the course of pediatric proton therapy besides the original CT simulation.

The verification of water equivalent path length deviation from the initial plan was conducted using the WEPL method. This procedure was executed on the original CT, rescanned CT, and synthetic CT images. The reference WEPL value was established based on the original CT images, serving as the benchmark. Subsequent WEPL values were derived from the synthetic CT images generated from the daily CBCT images and also the rescanned CT images. All three types of images—the original CT, rescanned CT, and synthetic CT—were integrated into the study’s dose calculations and plan quality assessments. Through this comprehensive approach, the study aimed to investigate the influence of anatomical variations on daily plan quality.

A comparative statistical analysis was undertaken to assess the efficacy of two distinct methods for validating WEPL: the original CT versus the rescanned CT, and the original CT versus the synthetic CT. To establish the commensurability of the synthetic CT images with the rescanned CT, a statistical *t*-test approach was employed. This method was utilized to ascertain whether the results produced by these methods are significantly similar or if there exists a noteworthy distinction between the two. The *p*-value, a key factor in this context, reflects the likelihood of obtaining test results that are equally or more extreme than the observed outcome, assuming the null hypothesis to be accurate. When there is no statistically significant difference between the compared groups, the *p*-value generally assumes a value exceeding the selected significance level (α). The significance level, determined prior to the statistical assessment, serves as a threshold for evaluating the outcomes of the test. A commonly employed significance level (α) is 0.05 (5%), denoting the threshold at which statistically significant findings are considered.

### 2.3. Treatment Planning Aspects

**Figure 2:**
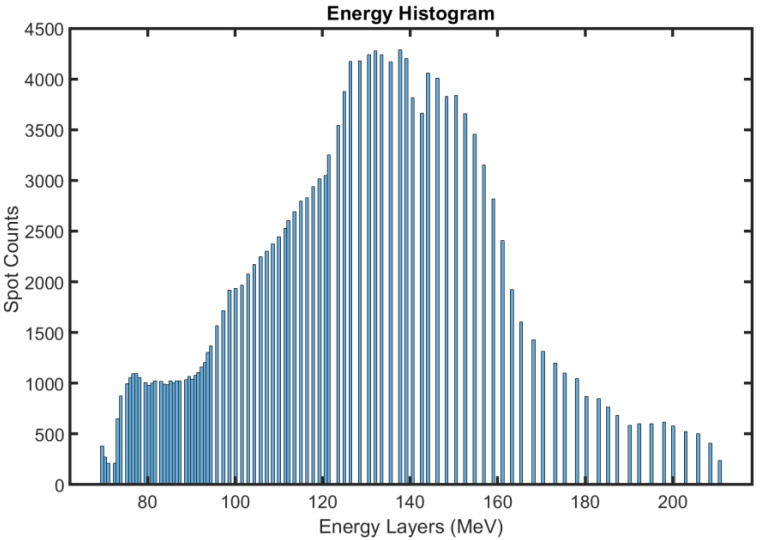
The histogram of the energy layers was displayed based on 10 patient cases from TPS.

The treatment plans of 10 patients were designed using pencil beam scanning proton beams from a commercial proton therapy system (PROBEAT-V, Hitachi America, Ltd., Santa Clara, CA, USA). Beam arrangements were comprised of two posterior-oblique beams to treat lower abdomen and pelvis tumors with a plan goal of CTV D95% = 95%. In brief, 3% range and 3 mm setup uncertainties were used for robust optimization. When large inter-fractional deviations were observed such as weight gain or loss, a replan was created based on the rescanned CT by using the same planning scheme and beam angles. Based on the treatment planning statistics, the number of proton beam energy layers and spots were 956 and 175,113, respectively as shown in Figure 2. The mean and SD of the energy layers were 121.1 MeV ± 30.3 MeV which corresponds to a 10.8 mm water equivalent distance. A total of 3% of range error in the planning was expected to compensate for 3 mm of range errors in terms of WEPL based on the average energy layers used in the treatment planning system.

## 3. Results

The WEPL deviations determined from the original CT to rescanned CT scans ranged from 0.1 to 12 mm at the target’s distal edge. A total of 5 out of 10 cases showed significant WEPL deviations from the original CT to the rescanned CT and the synthetic CT as an average of 10.4 mm and 9.8 mm, respectively. For these dramatic changes, protons’ spread-out Bragg peak (SOBP) regions were shifted and caused significant reductions in the dosimetric coverage of CTV Dmin as an average of 23.1% and 20.7% for the rescanned and synthetic CT plans, respectively. Statistical analysis on WEPL differences for all 10 cases between (original—rescanned CT) and (original—synthetic CT) revealed that the two methods were strongly correlated (r = 0.93) and showed no statistically significant difference (*p* = 0.81, α = 0.05), with the mean differences of −0.09 ± 1.65 mm.

Table 2 summarizes the patient cohort and outlines the WEPL differences for the distal edge of CTV between the original CT and the rescanned CT and the original CT and the synthetic CT. It reveals the impact on plan quality for CTV target coverage in terms of D95%, Dmin and Dmax parameters from the treatment planning.

**Figure 3:**
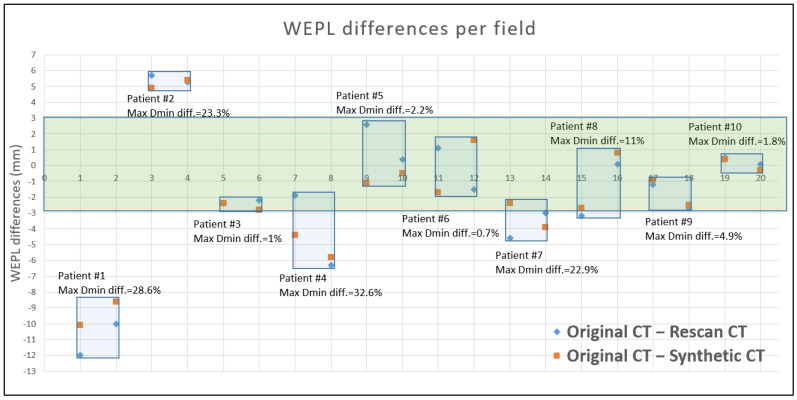
WEPL differences per field for the distal edge of CTV between the plans of original CT and rescanned CT and original CT and synthetic CT. Maximum Dmin differences from the original plans were annotated and a green shaded bar was drawn to indicate ±3 mm WEPL region.

Figure 3 demonstrates the WEPL differences per field for the distal edge of CTV between the plans of the original CT and the rescanned CT and the original CT and the synthetic CT with the annotation of maximum Dmin differences for CTV. It was determined that within ±3 mm water equivalent difference, the maximum Dmin deviations from the original plans were less than 5%, whereas, beyond of 3 mm WEPL difference, the CTV coverage significantly dropped for Dmin when compared to the original plans.

**Figure 4:**
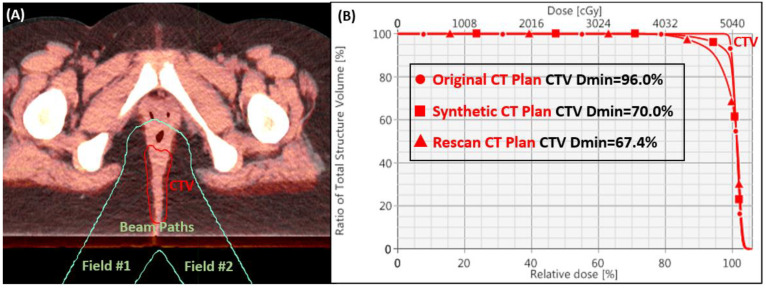
The tissue discrepancy of 12 mm was shown in the overlaid axial plane of the original and rescanned CTs in (**A**). The impact of plan quality was demonstrated for CTV Dmin in the DVH curves for original, rescanned, and synthetic CT plans in (**B**) for patient #1.

For fields #1 and #2 of the first patient, Figure 4 shows the transverse plane of the disease site where the rescanned CT was registered via bony anatomy and overlaid on the top of the original CT to emphasize the significant tissue discrepancy of 12 mm. Due to the weight gain, the proton’s SOBP was pulled back more posteriorly and caused catastrophic dose degradations revealed in the DVH curves of the CTV. The analysis of synthetic CT confirmed a 10.1 mm WEPL deviation from the distal edge of CTV from the original CT plan.

## 4. Discussion

Adaptive radiation therapy offers the promise of tailoring radiation treatment plans through a “plan of the day” approach, utilizing daily imaging from IGRT to ensure accurate delivery. However, several substantial barriers must be navigated for successful clinical integration of this approach. The complexity of workflow, involving daily imaging, plan adaptation, and quality assurance, can strain resources and necessitate efficient coordination between clinical teams. Generating treatment plans daily can lead to time and resource constraints, impacting schedules and increasing the workload for clinical staff. Rigorous quality assurance processes are vital to ensure the safety and accuracy of newly adapted plans. Dose accumulation and delivery verification methods must be developed to accurately assess the delivered dose against the planned dose over the course of treatment. Study [13] highlights the critical importance of fully characterizing the inherent errors and uncertainties within proton dose calculations and emphasizes the need to establish planning methods that robustly account for these factors. Efficient data management and IT infrastructure are also essential to handle the substantial amount of imaging and planning data generated. Decision-making algorithms that automatically determine when plan adaptation is necessary based on daily imaging data are among the challenges for a clinic that pursues adaptive radiation therapy.

Over the past decade, proton therapy facilities have transitioned from traditional 2D kV imaging to employing volumetric imaging systems such as CBCT or CT-on-rails for the purpose of image-guided proton therapy. This shift can be attributed to heightened commercial attention and the enhanced accessibility of volumetric imaging systems, in addition to the transition from passively scattered proton therapy to the adoption of intensity-modulated proton therapy [14]. Given the relatively recent introduction of in-room volumetric imaging within the context of proton therapy, it appears that the frequency of its utilization can exhibit variability across different treatment centers. Illustratively, a group [7] presents a practice wherein daily CBCT imaging is employed for verifying the daily positioning of pediatric patients including bony anatomy and patient volume changes. Conversely, another group [15] proposes a more limited employment of CBCT imaging, utilizing daily CBCT imaging for the initial five fractions and then reducing the frequency to twice weekly for their pediatric craniospinal irradiation patients. The variability in the approaches might be attributed to concerns about the additional imaging dose incurred by patients when transitioning from conventional 2D kV orthogonal imaging to 3D volumetric CBCT imaging. Consequently, it is imperative to carefully assess the supplementary cost against the potential benefits when determining the optimal frequency of volumetric imaging. When considering adaptive proton therapy, the significance of 3D volumetric CBCT imaging becomes apparent as it plays a vital role in quantifying anatomical changes within the body through synthetic CT images. These synthetic CT images can serve as surrogates to calculate the daily proton dose. Nevertheless, it is worth noting that the frequency of CBCT imaging can be fine-tuned, optimizing it according to the volatility in tumor volumes and the extent of interfractional variations based on the disease sites.

In this study, we applied proton interfractional range verification utilizing daily CBCT for pediatric patients with pelvic tumors. The daily proton range monitoring was able to reveal considerable variations in water equivalent variations that potentially could affect the clinical target volume coverage, highlighting the importance of proton range verification in daily clinical practice. Changes in the patient’s anatomy during the course of treatment, such as weight gain or loss, tumor progression, and regression, or changes due to surgery or other treatments, can affect WEPL estimations. WEPL estimation can be particularly useful in monitoring body circumference changes such as weight gain and loss in pediatric patients undergoing proton therapy due to homogeneous tissue expansion or contraction from the initial anatomy. Estimating WEPL can be challenging when protons pass through various tissues with different densities and compositions, such as the transition from soft tissue to bone or air-filled cavities like the lungs or sinuses [16]. This heterogeneity can lead to uncertainties in WEPL estimation and could affect the accuracy of the proton range. CBCT imaging modality may sometimes produce artifacts, which are inconsistencies or distortions in the images that do not correspond to actual anatomical structures [2]. Therefore, phantom validations are crucial prior to using WEPL and synthetic CT algorithms when estimating daily WEPL variations.

## 5. Conclusions

This study underscores the critical role of proton interfractional range verification based on daily CBCT in pediatric patients with pelvic tumors. By continually monitoring the WEPL, clinicians can adapt to dynamic anatomical changes and ensure optimal dose delivery. This is particularly crucial in a pediatric setting where physiological changes due to growth and development can influence the proton range significantly.

There remain challenges and concerns: the additional radiation exposure from the rescanned CT images warrants careful consideration given the lifetime impact of radiation exposure in pediatric patients. Technological advancements and improved protocols are needed to minimize exposure and CBCT-based WEPL monitoring may serve an important role in monitoring daily variations in anatomy.

These findings highlight the need for continuous improvement in WEPL estimation techniques and further optimization of daily CBCT-based proton range verification protocols. Balancing the benefits of enhanced treatment accuracy with the necessity to minimize treatment time and radiation exposure remains a pivotal concern in advancing pediatric proton therapy.

## Figures and Tables

**Table 1:** Summary of patient characteristics. Abbreviations: LPO, left posterior oblique; RPO, right posterior oblique; AP, anterior-posterior; RL, right lateral; PA, posterior-anterior; LL, left lateral; LPO, left posterior oblique; RAO, right anterior oblique.

Patient Number	Diagnoses	Age (Years)	Sex	Treatment Site	Dose Per Fraction (GyRBE) × Fraction	Total Dose (GyRBE)	Beam Orientations	Number of Rescans
1	Rhabdomyosarcoma	17	Female	Rectum	1.8 × 33	59.4	LPO, RPO	1
2	Fibrosarcoma	2	Male	Right Hip	1.8 × 30	54	AP, RL	2
3	Ewing’s Sarcoma	9	Female	Left Pelvis	1.8 × 36	64.8	LPO, RPO	1
4	Ewing’s Sarcoma	15	Female	Left Pelvis	1.8 × 36	64.8	PA, LL	3
5	Rhabdomyosarcoma	21	Male	Prostate	1.8 × 28	50.4	LL, RL	1
6	Rhabdomyosarcoma	15	Male	Left Sacrum	1.8 × 31	55.8	PA, LL	1
7	Rhabdomyosarcoma	17	Female	Left Perineum	1.8 × 33	59.4	LPO, LL	1
8	Sarcoma	23	Male	Left Pelvis	1.8 × 31	55.8	PA, LL	1
9	Ewing’s Sarcoma	13	Female	Right Ilium	1.8 × 36	64.8	PA, RAO	1
10	Ewing’s Sarcoma	16	Male	Sacrum	1.8 × 33	59.4	LPO, RPO	1

**Table 2:** 20 fields belonging to 10 patients were analyzed for the WEPL differences between original CT and rescanned CT and original CT and synthetic CT with the impact on plan quality in terms of CTV D95%, Dmin and Dmax.

Patient Number	Field Number	WEPL Diff. (mm) Original—Rescan	WEPL Diff. (mm) Original—Synthetic	CTV Dmin (%)	CTV D99% (%)	CTV D95% (%)	CTV Dmax (%)
				Original/Rescan/Synthetic	Original/Rescan/Synthetic	Original/Rescan/Synthetic	Original/Rescan/Synthetic
1	1	−12	−10.1	96/67.4/70.0	98.1/81.7/85.8	99.5/90.3/95.7	106/105.4/105.3
	2	−10	−8.6				
2	3	5.7	4.9	93/69.7/69.8	95.4/84.6/83.9	96.6/91.7/91.6	105.8/111.4/111.4
	4	5.3	5.4				
3	5	−2.4	−2.4	95.6/95.3/96	98.2/98.6/98.9	99.1/99.2/100	107.9/108.9/108.8
	6	−2.2	−2.8				
4	7	−1.9	−4.4	95/65.1/62.4	96.4/83.2/81.5	100/99.5/99.4	110.8/113.6/115.3
	8	−6.3	−5.8				
5	9	2.6	−1.1	95.3/94/94.4	97.4/96.3/95.2	96.4/96.1/95.8	100.3/101.5/102.5
	10	0.4	−0.5				
6	11	1.1	−1.7	97.7/97/97.5	99.0/98.4/98.9	100/100/99.9	105.9/105.5/105.8
	12	−1.5	1.6				
7	13	−4.6	−2.4	94.4/71.5/83.5	97.5/87.4/91.3	100/99/98	108/114.6/110.4
	14	−3	−3.9				
8	15	−3.2	−2.7	94.8/83.8/84.2	99.2/97.1/95.3	102/100/100	110.7/114.9/116.1
	16	0.1	0.8				
9	17	−1.2	−0.9	94.9/93/90	99.5/97.4/96.5	100/100/100	109.9/111.8/114.6
	18	−2.7	−2.5				
10	19	0.5	0.4	82.3/81.4/80.5	96.0/95.3/92.9	98.6/98.4/98.6	105.4/105.1/106.8
	20	0.1	−0.3				

## Data Availability

The data that support the findings of this study are available from the corresponding author, upon reasonable request.
